# Client preferences for HIV Care Coordination Program features in New York City: latent class analysis of a discrete choice experiment

**DOI:** 10.1002/jia2.26162

**Published:** 2023-08-29

**Authors:** Madellena Conte, Rebecca Zimba, Chunki Fong, Jennifer Carmona, Gina Gambone, McKaylee Robertson, Sarah Kozlowski, Faisal Abdelqader, Denis Nash, Mary Irvine

**Affiliations:** ^1^ Institute for Implementation Science in Population Health (ISPH) City University of New York (CUNY) New York New York USA; ^2^ Zucker School of Medicine at Hofstra/Northwell Hempstead New York USA; ^3^ New York City Department of Health and Mental Hygiene, Bureau of Hepatitis HIV and Sexually Transmitted Infections New York New York USA; ^4^ Department of Epidemiology and Biostatistics Graduate School of Public Health and Health Policy City University of New York (CUNY) New York New York USA

**Keywords:** adherence, care continuum, care coordination, DCE, HIV care, implementation science

## Abstract

**Introduction:**

The PROMISE study, launched in 2018, evaluates the implementation of revisions to the HIV Care Coordination Program (CCP) designed to minimize persistent disparities in HIV outcomes among high‐need persons living with HIV in New York City. We conducted a discrete choice experiment (DCE) assessing the preferences of CCP clients to inform improvements to the program's design.

**Methods:**

Clients chose between two hypothetical CCP options that varied across four program attributes: help with antiretroviral therapy (ART) adherence (directly observed therapy [DOT] vs. remind via phone/text vs. adherence assessment), help with primary care appointments (remind and accompany vs. remind and transport vs. remind only), help with issues other than primary care (coverage and benefits vs. housing and food vs. mental health vs. specialty medical care) and visit location (meet at home vs. via phone/video vs. program visit 30 or 60 minutes away). The latent class analysis identified different preference patterns. A choice simulation was performed to model client preferences for hypothetical CCPs as a whole.

**Results:**

One hundred and eighty‐one CCP clients from six sites implementing the revised CCP completed the DCE January 2020–March 2021. Most clients had stable housing (68.5%), reported no problem substance use in the last 3 months (72.4%) and achieved viral suppression (78.5) with only 26.5% receiving DOT within a CCP. 77.3% of responses were obtained before the COVID‐19 pandemic. Preferences clustered into three groups. Visit location and ART adherence support were the most important attributes. Group 1 (40%) endorsed telehealth for visit location; telehealth for ART adherence support; and help with securing housing/food; Group 2 (37%) endorsed telehealth for visit location; telehealth for ART adherence support; and staff reminding/arranging appointment transportation; Group 3 (23%) endorsed staff meeting clients at program location and staff working with clients for medication adherence. In the choice simulation, Basic and Medium hypothetical CCPs were endorsed more than Intensive CCPs.

**Conclusions:**

This DCE revealed a strong preference for telehealth and a relatively low preference for intensive services, such as DOT and home visits; preferences were heterogeneous. The findings support differentiated care and remote service delivery options in the NYC CCP, and can inform improvements to CCP design.

## 1 INTRODUCTION

Persistent inequities across the HIV care continuum threaten the United States’ progress towards ending the HIV epidemic [1]. In New York City, rates of viral load suppression (VS) are lower among transgender people, Black and Latino people, people experiencing housing instability and people with a history of injection drug use [2], disparities that persist in other epicentres [3] and nationwide [4]. To strengthen the HIV care continuum, the NYC Department of Health and Mental Hygiene (NYC Health Department) launched a multi‐component medical case management intervention known as the HIV Care Coordination Program (CCP) in 2009 to support people with HIV (PWH) at risk for, or with a recent history of, poor HIV outcomes [5]. The NYC CCP includes patient navigation, case management, directly observed therapy (DOT) and home visits, among other evidence‐based programmatic elements. The CCP led to substantial improvements in VS for participants newly diagnosed or consistently unsuppressed, and was designated by the US Centers for Disease Control and Prevention as a Structural Evidence‐Based Intervention [5, 6, 7, 8]. However, low levels of overall durable VS (<40%) underscored a need for intensive, sustained adherence support [7, 9, 10]. Based on identified implementation barriers and lessons learned from the early years of the program, the NYC Health Department undertook a substantial revision of the CCP model and a resolicitation of CCP service delivery contracts in 2017–2018. The PROMISE study (Program Refinements to Optimize Model Impact and Scalability based on Evidence) was launched in 2018 to assess the impact and implementation of the revised program model, as compared to the original CCP [11].

Aligning the design of program components with patient preferences can promote long‐term engagement, retention, and ultimately real‐world effectiveness [12]. An expanding body of research has solicited such preferences using discrete choice experiments (DCEs) [13]. A DCE is a quantitative technique commonly used in marketing research to identify and analyse relative preferences and evaluate trade‐offs between program components. DCEs have increasingly been utilized in healthcare research to inform the design and implementation of HIV care services [14, 15]. We report on the results of a DCE to understand the preferences of NYC CCP clients with regard to components of the revised CCP, including help with taking medication, help with primary care appointments, help with issues other than primary care and where program visits happen.

## 2 METHODS

### 2.1 Study setting, population and recruitment

Twenty‐five sites implemented the revised CCP. We recruited clients from a subset of six agencies partnering on the PROMISE study, purposively selected to represent diverse organizational settings and types of programs, and based on whether they had prior experience with the CCP. Four of the five NYC boroughs were represented among the six sites, and four of the six sites were clinic‐based. The only borough not represented (Staten Island) had the fewest CCP providers and the smallest prevalence of HIV. We focused on six partner sites for client DCE recruitment so that study team members could closely coordinate with site‐based liaisons on data collection. This ensured clients were informed of the DCE by service providers they knew, avoiding intrusion by the study team. Eligible participants included clients ≥18 years old currently enrolled in the CCP, as indicated by program staff reporting to the NYC Health Department via eSHARE (Electronic System for HIV/AIDS Reporting and Evaluation). A list of eligible clients was regenerated up to three times per agency throughout data collection to ensure the inclusion of clients enrolled in the CCP after the start of DCE recruitment. Study liaisons at the agencies were encouraged to make up to six outreach attempts per client. The six agencies participating in the client DCE included community health centres, community‐based organizations and a hospital, with locations in Manhattan, Queens, Brooklyn and the Bronx. The study was approved by the NYC Health Department Institutional Review Board, which is the IRB of record for the PROMISE study. All participants electronically provided informed consent.

### 2.2 Selection of attributes and levels

We previously detailed the development of the attributes and levels in the DCE [16]. In line with standard approaches to DCE design [17], we conducted focus group discussions with clients and providers from the partner sites to identify strengths and gaps in CCP services. We refined the wording of the attributes and levels based on discussions with PROMISE Study Advisory Board members. In developing the attributes, we aimed to include elements of the CCP amenable to future changes in terms of mode of delivery (i.e. visit location and methods of antiretroviral therapy [ART] adherence support) or program focus (i.e. level of emphasis on support for issues other than primary care).

### 2.3 DCE design

The DCE was designed with Lighthouse Studio Version 9.8.1 (Sawtooth Software). The final DCE included four attributes, each with 3–4 levels. Icons were included in the DCE to facilitate a comparison of the attribute levels (Table 1). Using Sawtooth's Balanced Overlap method, we generated an efficient DCE design with random choice tasks that were balanced (each attribute level appeared with equal frequency) and orthogonal (each level varied independently across attributes) [17]. The Balanced Overlap method facilitates identifying interactions between attributes, which we were interested in exploring. The final design had a relative D‐efficiency of 88% compared with Sawtooth's Completely Enumerated design. We presented clients with 10 choice tasks to minimize cognitive burden and maintain design efficiency [18]. Each choice task presented clients with two programs that differed by at least one level. We asked clients: “Imagine you had to choose between two programs with the features below. Select the one that you would prefer.” We chose not to include a “None” option to improve the precision of our main effects part‐worth utility estimates.

**Table 1:** Attributes and levels of a discrete choice experiment as displayed to clients taking the survey

Attribute description	Level description	Helper image
Help with taking medication	A staff member watches you take your medication	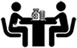
	You receive reminders by phone or text to take your medication	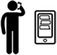
	You do not receive medication reminders, but a staff member works with you on sticking to a medication schedule	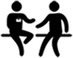
Help with primary care appointments	A staff member reminds you and goes with you to all primary care appointments	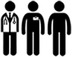
	A staff member reminds you and arranges transportation for you to get to your primary care appointments	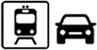
	A staff member only reminds you about your primary care appointments	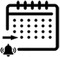
Help with issues other than primary care	Staff help with insurance, supplemental security income (SSI) benefits, and other general paperwork for healthcare coverage and benefits	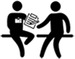
	Staff help with securing housing and food assistance	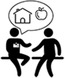
	Staff help with mental health and wellbeing issues (such as stress, substance use, diet or personal relationships)	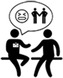
	Staff help with medical care from specialists (cardiologists, oncologists, neurologists, ear‐nose‐throat doctors, etc.)	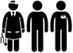
Where program visits happen	A staff member meets you in person at your home	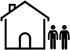
	A staff member meets you by phone or video chat	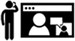
	A staff member meets you at a program location that is 30 minutes from your home	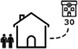
	A staff member meets you at a program location that is 60 minutes from your home	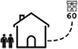

*Note*: Attribute and level descriptions may be abbreviated in the text and subsequent tables.

### 2.4 Recruitment target

We estimated 100 respondents to be the minimum number of responses to precisely estimate the main effects using the formula “*n* ≥ 500*c*/*ta*,” where “*n*” is the number of respondents, “*t*” is the number of choice tasks, “*a*” is the number of options per choice task and “*c*” is the largest number of levels for any attribute [19]. We aimed for 200 responses to estimate the main effects with greater precision and explore preference heterogeneity among participants.

### 2.5 Data collection

The DCE remained open from January 2020 until March 2021 to give agencies time to recruit participants during COVID‐19. The DCE was available in English, Spanish or French, and could be completed on any tablet with a modern browser. Site liaisons provided clients with the survey URL and login details either over the phone or in person when clients arrived at the site. Demo videos in English, Spanish and French were made available in October 2020 to support clients in self‐administering the survey and serve as tools for site liaisons when assisting clients. Clients received a $25 gift card for participation.

### 2.6 Analysis

#### 2.6.1 Client and agency characteristics

For analyses of client and agency characteristics, we used SAS software (Release 9.4 SAS Institute Inc.) and data from eSHARE. Calculating chi‐squared and Fisher's exact test *p*‐values, we compared the distribution of characteristics between the DCE participants and all clients who were enrolled in the CCP from 1 March 2020 to 28 February 2021 (a CCP contract year) and received at least one service during that period, and test for characteristics independence among the latent class analysis (LCA) group assignment. Statistical significance was evaluated at the 5% level.

#### 2.6.2 Relative importance and part‐worth utilities

We performed survey analysis using Sawtooth Software's Lighthouse Studio 9.8.1. We estimated part‐worth utilities for each level of each attribute, which represents the respondent's desirability for each attribute level. We calculated the relative importance for each attribute, which indicates the influence of each attribute relative to the other attributes on the client's overall decision‐making. For this analysis, we calculated the relative importance at the respondent level by dividing the range of part‐worth utilities for levels within an attribute by the sum of the ranges in all attributes. We averaged the respondent‐level value to yield an aggregate‐level estimate of the relative importance for each attribute [20].

#### 2.6.3 Latent class analysis

We used LCA to detect heterogeneous preference patterns according to the estimates of part‐worth utilities and relative importance [21]. To guide the selection of the number of latent class groupings, we compared model statistics for 2–5 group solutions, including Akaike's information criterion (AIC), Log‐likelihood ratio and the relative chi‐square difference. In addition to the above‐fit statistics, we also considered the number of participants per group and the interpretability and distinctness of the patterns of utilities between groups to decide on the final number of groups.

#### 2.6.4 Choice simulation analysis

Using utilities derived from the DCE, we used Sawtooth Software's Choice Simulator to model clients’ preference for hypothetical CCPs as a whole, to better mimic how clients would experience the CCP in the real world. Hypothetical CCPs were assembled by CCP quality management specialists from the NYC Health Department using the DCE attributes and levels (Table 2). We estimated “shares of preferences,” which represents the proportion of clients within each latent class that would endorse each hypothetical CCP. The randomized first‐choice model, which has a stronger predictive ability than other simulation models accounting for random error in point estimates of utilities, was used to generate preferences for hypothetical CCPs [20, 22]. In this model, utilities are summed across levels in each option and exponentiated to determine the probability an individual would endorse one hypothetical option over the others.

**Table 2:** Hypothetical CCPs with corresponding attribute levels

Hypothetical programs		Attribute‐level description			
Program type	Program subtype	Adherence support level	Primary care appointment level	Issues other than primary care level	Visit location level
**Basic medical case management**	**Basic‐1**	Medication assessment	Remind only	Specialty medical care	Phone or video chat
	**Basic‐2**	Medication reminders	Remind only	Specialty medical care	Phone or video chat
**Medium medical case management**	**Mid‐level‐1**	Medication reminders	Remind and accompany	General paperwork	Program visit 30 minutes away
	**Mid‐level‐2**	Medication reminders	Remind and accompany	General paperwork	Program visit 60 minutes away
	**Mid‐level‐3**	Medication reminders	Remind and accompany	Housing and food	Program visit 30 minutes away
	**Mid‐level‐4**	Medication reminders	Remind and accompany	Housing and food	Program visit 60 minutes away
**Intensive medical case management**	**Intensive‐1**	DOT	Remind and accompany	Mental healthcare	Meet at home
	**Intensive‐2**	DOT	Remind and accompany	Mental healthcare	Meet at home

#### 2.6.5 Sensitivity analysis: effect of “New York State on PAUSE” executive order

Beginning March 2020, all agencies transitioned to remote services and paused community‐based visits due to COVID‐19 and New York State on PAUSE order [23]. Some programs resumed community‐based visits summer of 2020, but most services remained remote. We analysed the timing of survey completion relative to service disruptions due to COVID‐19. Our hypothesis was that clients who completed the survey when in‐person services were paused would be more likely to have received telehealth services, which may have affected their preferences for telehealth in the DCE. We categorized responses as *pre‐pause* if they were submitted before the clients’ agency paused in‐person services, *intra‐pause* if submitted while in‐person services were suspended and *post‐pause* if submitted after in‐person services resumed. We assessed differences in distribution of survey timing among the three latent class groups with chi‐square.

## 3 RESULTS

### 3.1 Client and agency characteristics

Though the DCE could be remotely deployed, the pandemic‐related requirement to transition to remote CCP services in March 2020 posed a barrier to reaching the target sample size of 200 responses. Table 3 describes the characteristics of the 181 participants overall, by latent class group, and compared to the total population of enrolled CCP clients. For all clients completing the DCE, the median age was 53 years; 100 (55.3%) identified as cisgender men; 39 (76.8%) identified as straight or heterosexual; and 121 (66.9%) identified as Black. Most (*n* = 99, 54.7%) respondents reported a lifetime psychiatric diagnosis of depression or anxiety disorder; 41 (22.7%) reported problem substance use in the last 3 months; and 124 (68.5%) reported stable housing. Only 46 (25.4%) reported completing high school/GED and 106 (58.6%) reported they were unemployed. As of the date of their DCE, more than three‐quarters of respondents (*n* = 142, 78.5%) had viral load suppression (<200 copies/μl) and 84 (46.4%) had CD4 ≥ 500 cells/mm^3^. Most clients had been enrolled in a CCP for more than 1 year (*n* = 167, 92.3%) and only 48 (26.5%) received DOT within a CCP. Analysis comparing the DCE participants to all clients enrolled in the CCP found statistically significant differences for the following characteristics: self‐reported gender (more cisgender women, fewer cisgender men than CCP), sexual identity (more heterosexual, fewer non‐binary than CCP), race/ethnicity (more Black, fewer White and Hispanic than CCP), psychiatric diagnosis, length of time enrolled in care coordination and employment status (fewer employed than CCP).

**Table 3:** Discrete choice experiment client characteristics, by latent class group

				Latent class groups			
Client characteristic	All clients enrolled in CCP^a^ *N* = 3395 *n* (%)	DCE participants *N* = 181 *n* (%)	DCE participants versus all CCP clients *p*‐value***	Group 1 *n* = 72 *n* (%)	Group 2 *N* = 67 *n* (%)	Group 3 *n* = 42 *n* (%)	Latent class group comparison *p*‐value**
**Age**							
Median [IQR]	51 [37–60]	53 [42–61]	–	56 [43–61.5]	53 [41–61]	53 [44–60]	–
**Age category**							
<40	996 (29.3)	38 (21.0)	0.102	14 (19.4)	15 (22.4)	9 (21.4)	0.756
40–49	619 (18.2)	34 (18.8)		10 (13.9)	16 (23.9)	8 (19.1)	
50–59	878 (25.9)	55 (30.4)		24 (33.3)	17 (25.37)	14 (33.3)	
>60	902 (26.6)	54 (29.8)		24 (33.3)	19 (28.4)	11 (26.2)	
**Self‐reported gender**							
Cisgender man	2094 (61.7)	100 (55.3)	0.002	41 (56.9)	41 (61.2)	18 (42.9)	0.187
Cisgender woman	1140 (33.6)	80 (44.2)		30 (41.7)	26 (38.8)	24 (57.1)	
Transgender, non‐binary or gender non‐conforming	145 (4.2)	1 (0.6)		1 (1.4)	0 (0.0)	0 (0.0)	
Unknown	16 (0.5)	0 (0.0)		0 (0.0)	0 (0.0)	0 (0.0)	
**Sexual identity**							
Homosexual, bisexual, pansexual or queer	1195 (35.2)	40 (22.1)	<0.001	14 (19.4)	19 (28.4)	7 (16.7)	0.466
Straight or heterosexual	2064 (60.8)	139 (76.8)		57 (79.2)	47 (70.2)	35 (83.3)	
Unknown/missing	136 (4.0)	2 (1.1)		1 (1.4)	1 (1.5)	0 (0.0)	
**Race/ethnicity**							
Hispanic/Latinx^b^	1349 (39.7)	51 (28.2)	0.002	18 (25.0)	18 (26.9)	15 (35.7)	0.799
American Indian/Alaska Native, Asian/Pacific Islander, Multiracial	120 (3.5)	4 (2.2)		2 (2.8)	1 (1.5)	1 (2.4)	
Black	1751 (51.6)	121 (66.9)		49 (68.1)	46 (68.7)	26 (61.9)	
White	153 (4.5)	5 (2.8)		3 (4.2)	2 (3.0)	0 (0.0)	
Unknown	22 (0.7)	0 (0.0)		0 (0.0)	0 (0.0)	0 (0.0)	
**Psychiatric diagnosis (lifetime history)**							
Depression or anxiety disorder	1543 (45.5)	99 (54.7)	0.015	43 (59.7)	32 (47.8)	24 (57.1)	0.344
Psychosis (e.g. schizophrenia) or bipolar disorder	499 (14.7)	41 (22.7)	0.004	16 (22.2)	13 (19.4)	12 (28.6)	0.535
PTSD	273 (8.0)	16 (8.8)	0.701	5 (6.9)	7 (10.5)	4 (9.5)	0.756
Other	216 (6.4)	5 (2.8)	0.05	1 (1.4)	4 (6.0)	0 (0.0)	0.208
None	1553 (45.7)	66 (36.5)	0.015	22 (30.6)	30 (44.8)	14 (33.3)	0.196
**Problem substance use in the last 3 months** c							
Yes	796 (23.5)	41 (22.7)	0.994	18 (25.0)	11 (16.4)	12 (28.6)	0.241
No	2540 (74.8)	131 (72.4)		49 (68.1)	52 (77.6)	30 (71.4)	
Unknown	59 (1.7)	9 (5.0)		5 (6.9)	4 (6.0)	0 (0.0)	
**Housing type** d							
Stable	2344 (69.0)	124 (68.5)	0.875	50 (69.4)	46 (68.7)	28 (66.7)	0.953
Unstable	1050 (30.9)	57 (31.5)		22 (30.6)	21 (31.3)	14 (33.3)	
Unknown	1 (<0.1)	0 (0.0)		0 (0.0)	0 (0.0)	0 (0.0)	
**Highest level of schooling completed**							
Below high school/GED	2443 (72.0)	135 (74.6)	0.483	59 (81.9)	45 (67.2)	31 (73.8)	0.134
High school/GED or above	941 (27.7)	46 (25.4)		13 (18.1)	22 (32.8)	11 (26.2)	
Unknown	11 (0.3)	0 (0.0)		0 (0.0)	0 (0.0)	0 (0.0)	
**Receipt of directly observed therapy (DOT) within a care coordination program** e							
Yes	837 (24.7)	48 (26.5)	0.571	21 (29.2)	19 (28.4)	8 (19.1)	0.454
No	2558 (75.3)	133 (73.5)		51 (70.8)	48 (71.6)	34 (80.9)	
**Length of time enrolled in care coordination (CCP and CCR)**							
< 1 month	7 (0.2)	0 (0.0)	<0.001	0 (0.0)	0 (0.0)	0 (0.0)	0.154
1–6 months	407 (12.0)	1 (0.6)		1 (1.4)	0 (0.0)	0 (0.0)	
6–12 months	614 (18.1)	13 (7.2)		2 (2.8)	6 (9.0)	5 (11.9)	
> 1 year	2367 (69.7)	167 (92.3)		69 (95.8)	61 (91.0)	37 (88.1)	
**Employment status**							
Employed	608 (17.9)	17 (9.4)	<0.001	6 (8.3)	8 (11.9)	3 (7.1)	0.451
Unemployed	2184 (64.3)	106 (58.6)		39 (54.2)	41 (61.2)	26 (61.9)	
Out of workforce	556 (16.4)	56 (30.9)		27 (37.5)	16 (23.9)	13 (31.0)	
Unknown	47 (1.4)	2 (1.1)		0 (0.0)	2 (3.0)	0 (0.0)	
**Viral load category**							
Suppressed (< 200 copies/ml)	2496 (73.5)	142 (78.5)	0.142	57 (79.2)	53 (79.1)	32 (76.2)	0.921
Unsuppressed (≥ 200 copies/ml)	899 (26.5)	39 (21.5)		14 (20.8)	14 (20.9)	10 (23.8)	
**CD4 category**							
< 200 cells/mm3	667 (19.7)	34 (18.8)	0.502	18 (25.0)	8 (11.9)	8 (19.1)	0.438
200–349 cells/mm3	611 (18.0)	26 (14.4)		11 (15.3)	9 (13.4)	6 (14.3)	
350–499 cells/mm3	589 (17.4)	37 (20.4)		14 (19.4)	17 (25.4)	6 (14.3)	
≥ 500 cells/mm3	1526 (45.0)	84 (46.4)		29 (40.3)	33 (49.3)	22 (52.4)	
Unknown	2 (0.1)	0 (0.0)		0 (0.0)	0 (0.0)	0 (0.0)	
**Timing of DCE completion** f							
Pre‐pause, before the COVID‐19 pandemic		140 (77.3)	–	38 (90.5)	55 (76.4)	47 (70.1)	0.053
Intra‐pause, while in‐person services were paused		15 (8.3)		1 (2.4)	9 (12.5)	5 (7.5)	
Post‐pause, after in‐person services resumed		26 (14.4)		3 (7.1)	8 (11.1)	15 (22.4)	

^a^ Includes all clients who were enrolled in CCP from 1 March 2020 to 28 February 2021 and received at least one service during that period.

^b^ Includes all clients with that reported ethnicity, regardless of race.

^c^ Recent report of binge drinking, heroin, meth, cocaine or recreational prescription drug use in the last 3 months. Reported as of client's latest available eSHARE assessment before the date of survey completion.

^d^ “Stable” defined as any permanent housing situation. “Unstable” defined as any temporary/transitional housing situation or being homeless. Reported as of client's latest available eSHARE assessment before the date of survey completion.

^e^ Includes DOT received from CCP in the last decade.

^f^ Range of pause start dates: 3/9/2020–4/15/2020, range of resumption dates: 4/30/2020–11/1/2020.

** *p*‐Values represent significance testing performed to compare sample group membership for each characteristic.

*** *p*‐Values represent significance testing performed to compare the total sample with all CCR clients for each demographic characteristic.

The timing of pauses and resumptions of in‐person services varied by partnering CCP agency. Overall, 77.3% of surveys were completed pre‐pause, and 8.3% were completed intra‐pause, and 14.4% were completed post‐pause. Differences in distribution of survey timing among the three classes overall is significant at 0.053. Pairwise comparisons suggest the difference between Groups 2 and 3 is statistically significant (chi‐square *p*‐value = 0.045, Fisher's exact *p*‐value = 0.035), but not between Groups 1 and 3 nor Groups 1 and 2.

Table 4 describes clients by agency characteristics, overall and by latent class. There was a statistically significant difference in the proportion of clients enrolled in clinic‐based CCP programs between groups, with 73.1% of clients in Group 2 from a clinic‐based agency, cs 64.3% in Group 3 and 51.4% in Group 1. See also Table S1 for additional details on site characteristics.

**Table 4:** Clients by agency characteristic and latent class group

Agency characteristic (*n* of agencies)		Latent class groups			Latent class group comparison *p*‐value
	Total *N* = 181 *n* (%)	Group 1 *n* = 72 *n* (%)	Group 2 *n* = 67 *n* (%)	Group 3 *n* = 42 *n* (%)	
**Agency type**					0.029
Clinic‐based (4)	113 (62.4)	37 (51.4)	49 (73.1)	27 (64.3)	
Non‐clinic (2)	68 (37.6)	35 (48.6)	18 (26.9)	15 (35.7)	
**Borough of agency**					0.689
Bronx (2)	69 (38.1)	30 (41.7)	22 (32.8)	17 (40.5)	
Brooklyn (1)	38 (21.0)	16 (22.2)	15 (22.4)	7 (16.7)	
Manhattan (2)	41 (22.7)	17 (23.6)	14 (20.9)	10 (23.8)	
Queens (1)	33 (18.2)	9 (12.5)	16 (23.9)	8 (19.0)	
**Care coordination program experience**					0.214
Experience with the initial and revised program (3)	81 (44.8)	36 (50.0)	31 (46.3)	14 (33.3)	
New to care coordination under the revised program (3)	100 (55.2)	36 (50.0)	36 (53.7)	28 (66.7)	

### 3.2 Latent class group segmentation

We ran 2‐ to 5‐group LCA solutions. Although the 5‐class solution had a higher Log‐likelihood and lower AIC, we ultimately selected a 3‐class solution because of the higher chi‐square difference relative to the 2‐class solution, to retain classes of relatively larger size than higher‐order solutions, and for a more interpretable set of preference patterns. See Table S2 for model fit statistics. The 3‐class solution yielded 72 members (40%) in Group 1, 67 members (37%) in Group 2 and 42 members (23%) in Group 3. No client characteristic was significantly associated with group membership (Table 3). Agency type was significantly associated with group membership (Table 4).

### 3.3 Relative importance and part‐worth utilities

**Figure 1:**
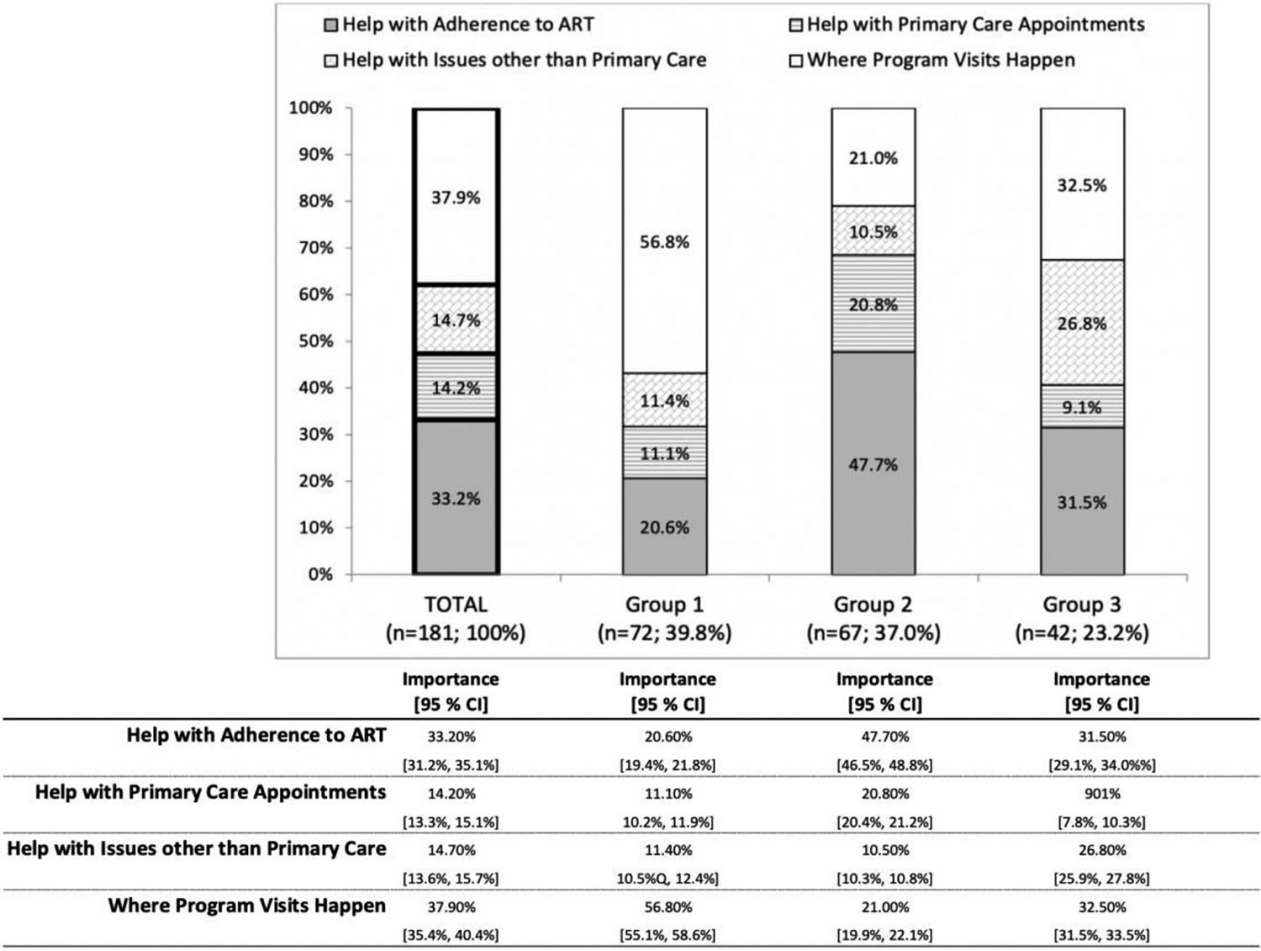
Relative importance of features of the HIV Care Coordination Program (CCP) among CCP clients, by latent class grouping.

For all participants, the attribute with the highest average relative importance was *Where Program Visits Happen* (37.9%), followed by *Help with Adherence to ART* (33.2%), *Help with Issues Other than Primary Care* (14.7%), and, lastly, *Help with Primary Care Appointments* (14.2%) (Figure 1). Overall, meeting a staff member by phone or video chat had the highest utility for *Where Program Visits Happen* (30.37 [95% CI 22.12, 38.61]); receiving reminders by phone or text to take medication had the highest utility for *Help with Adherence to ART* (46.34 [42.23, 50.44]); staff helping with securing housing and food assistance had the highest utility for *Help with Issues Other than Primary Care* (22.96 [17.85, 28.07]); and staff members reminding and arranging transportation for clients to get to primary care appointments had the highest utility for *Help with Primary Care Appointments* (20.69 [17.36, 24.02]). No interactions were identified between attributes. See also Table 5.

**Table 5:** Part‐worth utilities of CCP features, by latent class grouping

Attributes and levels	Total (*n* = 181) Utility (95% CI)	Latent class groups		
		Group 1 (*n* = 72) Utility (95% CI)	Group 2 (*n* = 67) Utility (95% CI)	Group 3 (*n* = 42) Utility (95% CI)
**Help with adherence to ART**				
Directly observed therapy	−57.60 (−65.99 −49.21)	2.37 (−5.24, 9.98)	−113.33 (−117.15, −109.50)	−71.51 (−79.01, −64.00)
Reminder via phone or text	46.34 (42.23, 50.44)	34.05 (30.77, 37.33)	77.40 (76.57, 78.22)	17.85 (11.57, 24.13)
Adherence assessment	11.26 (5.07, 17.45)	−36.42 (−40.90, −31.94)	35.93 (32.77, 39.08)	53.66 (50.71, 56.61)
**Help with primary care appointments**				
Remind and accompany	−20.87 (−24.26, −17.47)	−20.86 (−22.66, −19.05)	−42.87 (−43.73, −42.01)	14.20 (10.04, 18.37)
Remind and arrange transportation	20.69 (17.36, 24.02)	23.50 (21.90, 25.10)	40.37 (39.50, 41.25)	−15.53 (−19.59, −11.46)
Remind only	0.18 (−0.19, 0.55)	−2.64 (−2.92, −2.37)	2.49 (2.31, 2.68)	1.32 (1.09, 1.56)
**Help with issues other than primary care**				
Insurance, SSI benefits and other paperwork	−13.52 (−14.88, −12.17)	−15.00 (−16.00, −14.00)	−4.14 (−5.12, −3.15)	−25.97 (−26.95, −24.99)
Securing housing and food	22.96 (17.85, 28.07)	21.06 (17.86, 24.25)	−8.96 (−11.88, −6.03)	77.13 (73.93, 80.33)
Mental health and wellbeing	−19.73 (−20.91, −18.55)	−21.49 (−22.53, −20.44)	−11.84 (−12.91, −10.77)	−29.29 (−30.60, −27.98)
Connections to specialty medical care	10.29 (7.48, 13.10)	15.43 (14.18, 16.68)	24.93 (23.96, 25.91)	−21.87 (−24.74, −19.00)
**Where program visits happen**				
At home	14.18 (7.79, 20.57)	60.57 (56.05, 65.08)	−31.20 (−34.46, −27.94)	7.05 (2.48, 11.63)
Via phone or video chat	30.37 (22.12, 38.61)	79.74 (76.49, 82.99)	34.05 (30.42, 37.68)	−60.15 (−67.02, −53.28)
At program location, 30 minutes from home	25.46 (22.02, 28.90)	5.64 (2.92, 8.36)	24.00 (21.82, 26.18)	61.79 (60.50, 62.77)
At program location, 60 minutes from home	−70.01 (−79.75, −60.27)	−145.94 (−150.76, −141.12)	−26.85 (−32.13, −21.58)	−8.69 (−17.86, 0.48)

Abbreviation: SSI, supplemental security income.

### 3.4 Preferences for CCPs

**Figure 2:**
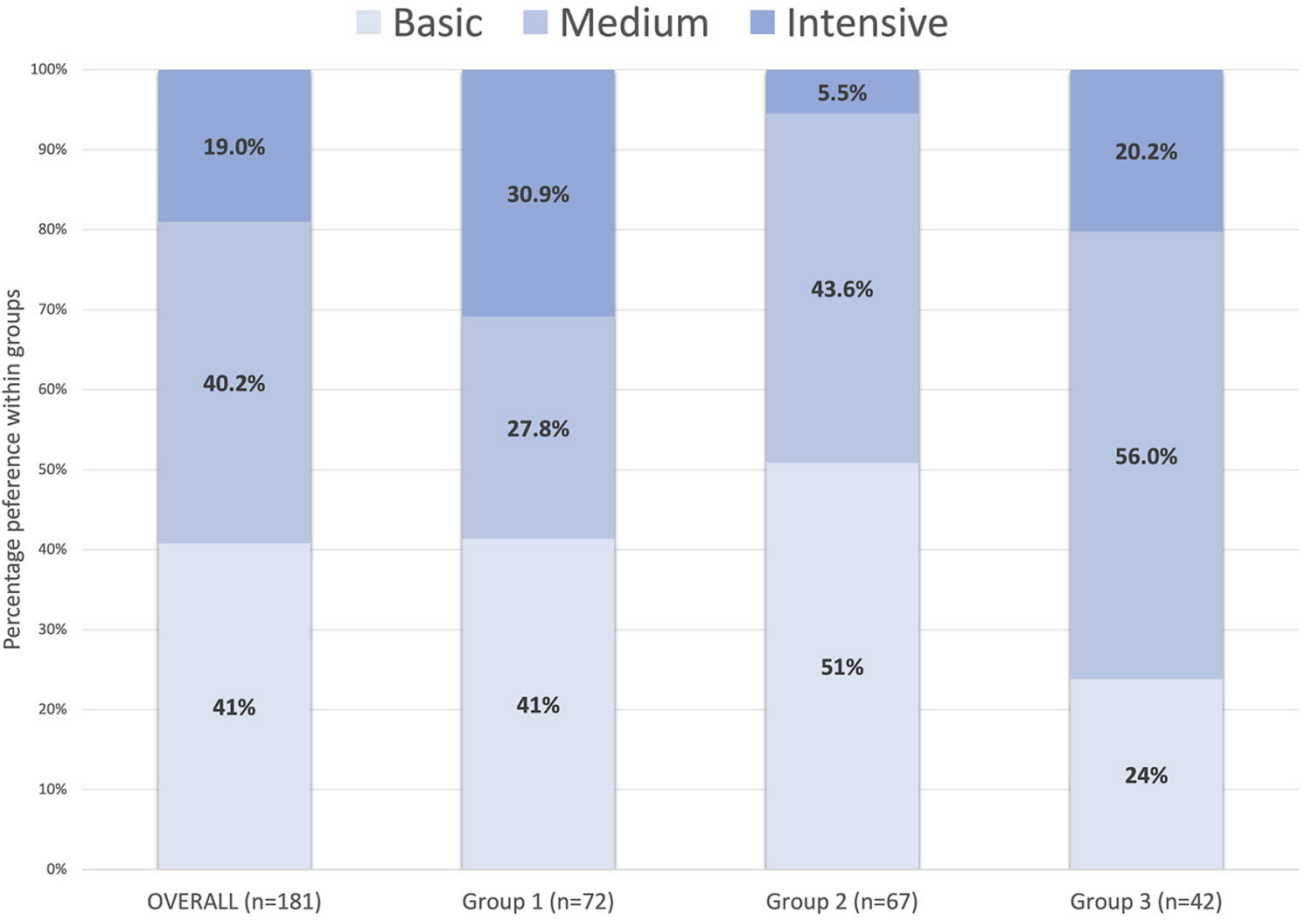
Plot of simulated shares of preference for medical case management service intensity by latent class group.

For simplicity, the shares of preference for the eight hypothetical programs were aggregated into three program types defined by medical case management service intensity as shown in Table 2: Basic, Medium and Intensive medical case management. Overall, client preference for Basic and Medium medical case management were 41% and 40%, respectively, with 19% endorsement of Intensive medical case management. See Figure 2 for the shares of preference for hypothetical CCPs overall and by group.

### 3.5 Sensitivity analysis

The relative importance from the sensitivity analysis by survey completion timing changed the most for *Help with Adherence to ART* (from 30.2% in the intra‐pause era to 38.6% in the post‐pause era) and *Where Program Visits Happen* (from 30.7% in the post‐pause era to 44.1% in the intra‐pause era) (Figure S1). The biggest differences in utilities were for DOT (−39.2 in the intra‐pause era to −82.3 in the post‐pause era) and for meeting at a program location 60 minutes from home (−96.7 in the intra‐pause era to −52.1 in the post‐pause era). The magnitude but not the direction of preferences changed depending on timing, with two notable exceptions: clients who completed the DCE in the intra‐pause period had negative utilities for meeting at home or at the program location 30 minutes from home, whereas in all other eras, the utilities for these levels were positive. See Figures S1 and S2 for the results of the sensitivity analysis. The numbers of participants in the intra‐ and post‐pause eras were small, and no hypothesis testing was performed.

## 4 DISCUSSION

Aligning program design with patient preferences, elicited in a DCE, can increase uptake, engagement and retention in care [12]. This DCE revealed two key general preferences among clients in an HIV CCP; clients endorsed telehealth for ART adherence support and CCP visit location, and clients endorsed intensive services, such as DOT and CCP home visits, comparatively less frequently than less intensive CCP services. Our analysis identified three distinct groups with heterogeneous preferences: Group 1 (40%) endorsed telehealth for CCP visit location and ART adherence support and endorsed help with securing housing/food; Group 2 (37%) endorsed telehealth for CCP visit location and ART adherence support and endorsed staff reminding and arranging transportation to get to primary care appointments; Group 3 (23%) endorsed staff meeting clients at program location for CCP visits (30 minutes from clients’ home) and staff working with clients to stick to medication schedules.

Clients endorsed telehealth‐focused care for visit location (staff meeting clients by phone/video) and ART adherence support (reminders via phone/text), two attributes with the highest relative importance among all participants and across the three subgroups identified by LCA. For *visit location*, all participants and clients in the two largest sub‐groups (Groups 1 and 2) endorsed CCP staff meeting clients by phone or video chat. This strength of the endorsement of telehealth may be related to the timing of survey completion relative to COVID‐19. While most clients in all latent class groups completed the DCE pre‐pause, Groups 1 and 2 had higher proportions of post‐pause responses and thus include the most members whose responses may have been influenced by changes in service delivery due to COVID‐19. Group 2, which had the largest negative utility for CCP staff meeting clients at their home, had the largest proportion of post‐pause responses. In contrast, Group 3, which endorsed staff members meeting clients at the program location, had the highest proportion of pre‐pause responses, and thus had fewer members whose responses may have been influenced by COVID‐19. Though the COVID‐19 pandemic undeniably has changed the way healthcare and support services are delivered, and may have increased the adoption of virtual options, there was already a positive preference for telehealth among responses submitted before the pandemic. This finding builds on prior research of preferences among PWH, which have shown endorsement for provider continuity and additional clinic locations [24]. The present study supports telehealth as a preferred option for CCP services, particularly with the accelerated adoption of telemedicine during the pandemic [25, 26]. However, this modality may not be appropriate for clients with unstable housing or other barriers to telehealth, who often face compounded socio‐economic and medical challenges, including barriers to accessing phones and maintaining phone service [27].

Second, this DCE highlighted a relatively low endorsement of intensive services, such as DOT and home visits, compared with other CCP services. Importantly, DCEs elicit trade‐offs; a low relative utility of one CCP level does not equate with a lack of value but rather reflects an endorsement of other CCP services. For example, all participants and Groups 1 and 2 preferred receiving reminders by phone/text over DOT for *Help with Adherence to ART* and preferred meeting staff via phone/video over home visits for *Where Program Visits Happen*. This pattern was also reflected in the choice simulation; 19% of all participants endorsed Intensive hypothetical CCPs, compared with 41% and 40% for Basic and Medium hypothetical CCPs, respectively. Client characteristics likely influenced preferences for services, in that most clients had stable housing, reported no problem substance use in the last 3 months and achieved VS (without DOT), similar to all clients enrolled in CCP during the period. The fact that most clients had achieved VS (78.5%), with only 26.5% receiving DOT within a CCP, is a potential explanation for the relatively low endorsement of DOT in the DCE.

There are limitations to this study. DCEs are based on stated preferences, not client behaviour; our results may not be predictive of actual behaviour. However, stated and revealed preferences were found to agree in a systematic review [28]. Second, though we met the minimum sample size for main effects, the pause of in‐person services due to COVID‐19 was a barrier to reaching our target of 200 responses, which could have limited our ability to detect preference differences between groups. Third, while sampling included clients from six sites selected to reflect the diversity of settings and care coordination experience of the sites implementing the revised CCP, the preferences elicited in the DCE do not necessarily represent the preferences of all clients in the CCP or PWH in NYC at large.

Clients who completed a DCE were comparable to all clients enrolled in the CCP across most characteristics. However, 92.3% of clients who completed the survey had been receiving care coordination support for over a year compared with 69.7% for all clients enrolled in CCP, a statistically significant difference that may have influenced responses. Staff may have had more success recruiting longer‐term clients to participate in the study than shorter‐term clients. These longer‐term clients may have achieved more stability in their care and may have been more likely to endorse less intensive services compared to clients newer to the program. Future work could attempt to recruit more newly enrolled clients to better understand the preferences of that population.

## 5 CONCLUSIONS

The findings from this client DCE support a client‐centred approach to adjusting the intensity of services and remote service delivery options in the NYC CCP. In the revisions to the CCP, which the PROMISE study was designed to evaluate, the NYC Health Department adjusted the service standards to allow for more differentiated care and telehealth versions of CCP services; these findings reinforce the value of those changes. Further research is needed to explore the concordance of preferences for CCP features between clients and service providers.

## AUTHORS’ CONTRIBUTIONS

MC led manuscript writing and development. RZ, CF, FA and MC conducted data analysis and synthesized results. JC and GG provided scientific insight for the DCE design, analysis and interpretation of results, including the choice experiment. MI, DN, SK and MR led the study design and implementation, defined the manuscript scope and contributed to the manuscript. DN, MI, SK, MI, JC and GG contributed to the study conceptualization, provided scientific oversight, and contributed to data interpretation and manuscript review.

## COMPETING INTERESTS

No competing interests for the authors in this study.

## FUNDING

This work was supported by the NIH PROMISE study. Grant number R01 MH117793. Project Officer Christopher M Gordon, cgordon1@mail.nih.gov

## Supporting information


**Table S1**. Partnering CCP site characteristics
**Table S2**. Comparison of LCA model fit statistics by number of classes
**Figure S1**. Relative importance of features of the HIV CCP among CCP clients, by timing of survey completion
**Figure S2**. Utilities for features of the HIV CCP among CCP clients, by timing of survey completion

## Data Availability

The data that support the findings of this study are available from the corresponding author upon reasonable request.
