# Supplementary material for: Client preferences for HIV Care Coordination Program features in New York City: latent class analysis of a discrete choice experiment
Source: J Int AIDS Soc. 2023 Aug 29;26(8):e26162. doi: 10.1002/jia2.26162 (PMC10465014; doi:10.1002/jia2.26162)
Supplement: Supplementary file 1 — Table S1. Partnering CCP site characteristics Table S2. Comparison of LCA model fit statistics by number of classes Figure S1. Relative importance of features of the HIV CCP among CCP clients, by timing of survey completion Figure S2. Utilities for features of the HIV CCP among CCP clients, by timing of survey completion [file JIA2-26-e26162-s001.docx]

Supplemental materials for “Client Preferences for HIV Care Coordination Program Features in New York City: Latent Class Analysis of a Discrete Choice Experiment”

**Supplemental Table 1. Partnering CCP site characteristics**

| **Agency code** | **Borough** | **CCP-experienced vs. new** | **Clinic-based vs. Non-clinic-based** |
| --- | --- | --- | --- |
| A | Queens | New | Clinic-based |
| B | Bronx | New | Clinic-based |
| C | Manhattan | New | Non-clinic |
| D | Brooklyn | Experienced | Clinic-based |
| E | Bronx | Experienced | Non-clinic |
| F | Manhattan | Experienced | Clinic-based |

**Supplemental Table 2. Comparison of LCA model fit statistics by number of classes**

| Class # | AIC | Log likelihood | Chi-square | Chi-square difference |
| --- | --- | --- | --- | --- |
| 2 | 2366.87 | -1162.43 | 142.74 | ----- |
| ***3*** | ***2361.90*** | ***-1148.95*** | ***169.70*** | ***26.96*** |
| 4 | 2358.17 | -1136.09 | 195.43 | 25.73 |
| ***5*** | ***2356.08*** | ***-1124.04*** | ***219.52*** | ***24.09*** |

**Supplemental Figure 1. Relative importance of features of the HIV CCP among CCP clients, by timing of survey completion**

**
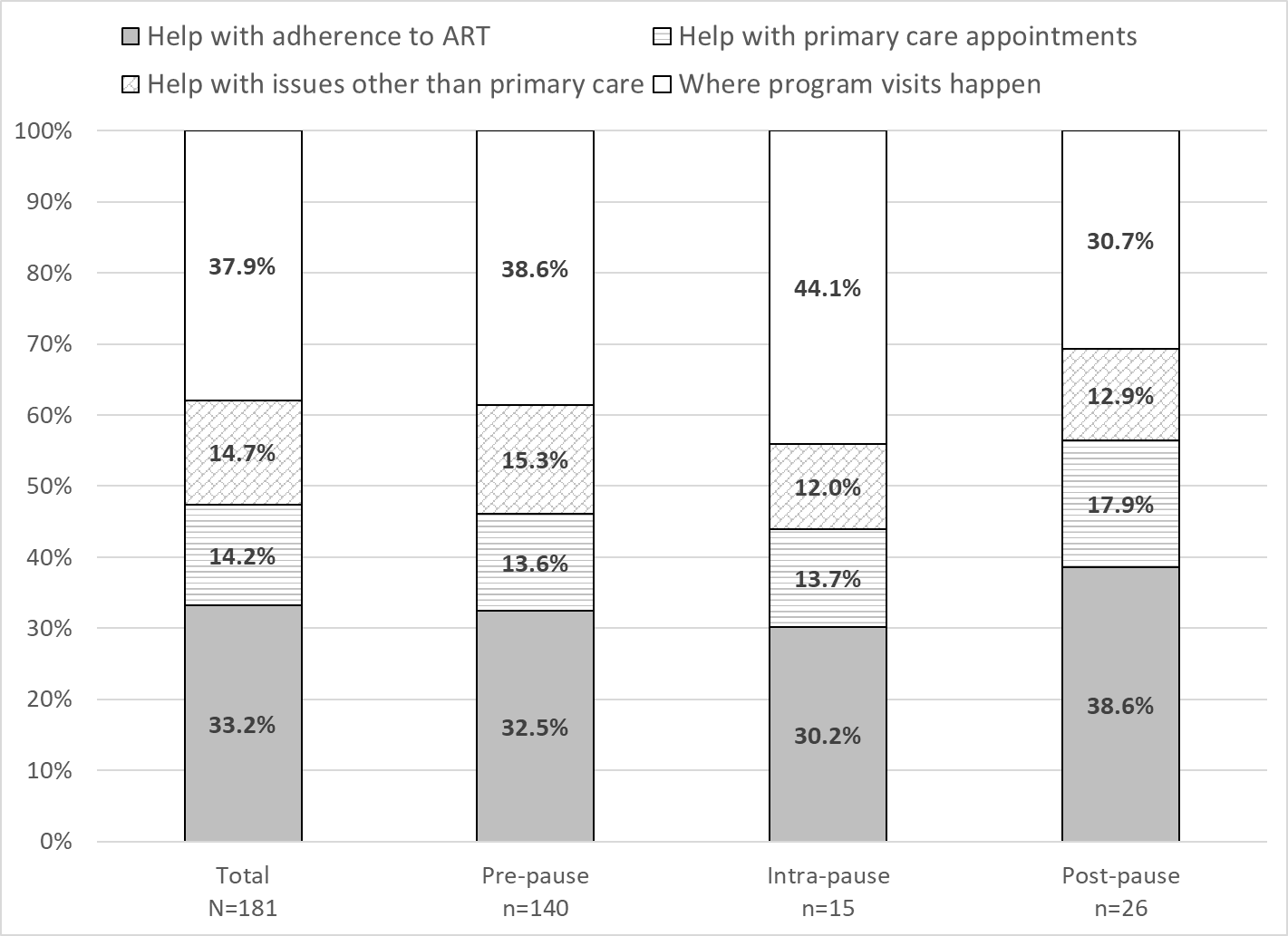
**

**Supplemental Figure 2. Utilities for features of the HIV CCP among CCP clients, by timing of survey completion**

**
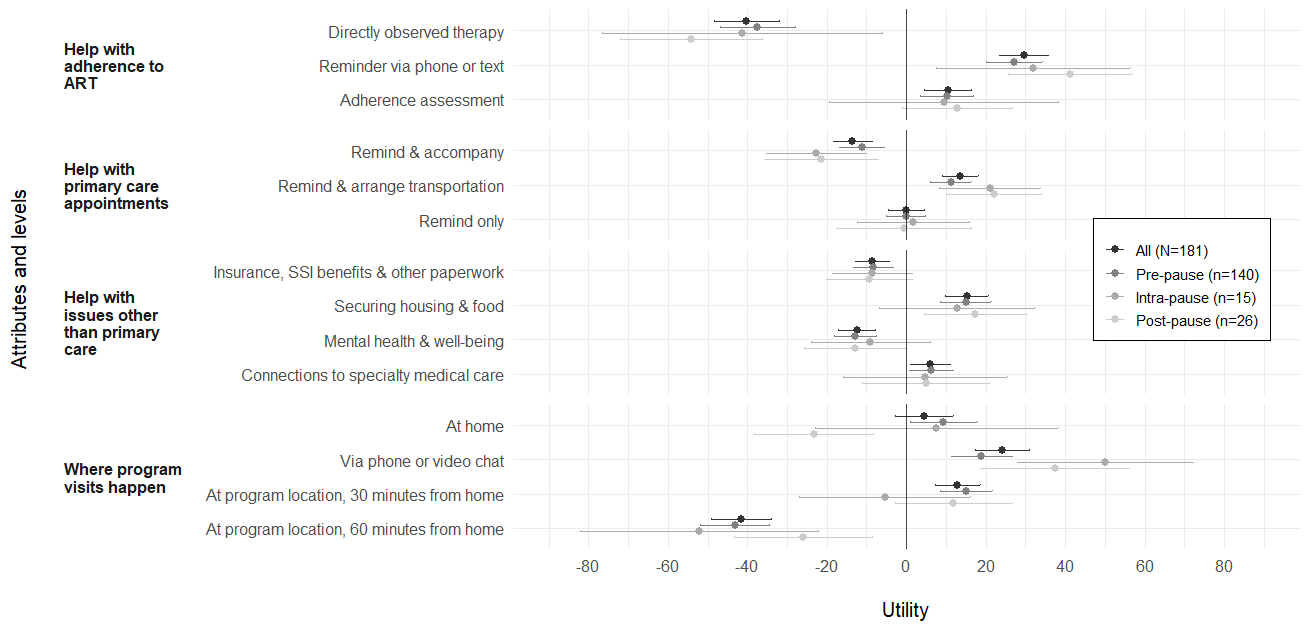
**

SSI = Supplemental security income
